# Research progress on the mechanism of ferroptosis and its role in diabetic retinopathy

**DOI:** 10.3389/fendo.2023.1155296

**Published:** 2023-06-01

**Authors:** Wei He, Lu Chang, Xinlu Li, Yan Mei

**Affiliations:** ^1^ Faculty of Life Science and Technology, Kunming University of Science and Technology, Kunming, China; ^2^ Department of Ophthalmology, The Affiliated Hospital of Kunming University of Science and Technology, Kunming, China; ^3^ Department of Ophthalmology, The First People's Hospital of Yunnan Province, Kunming, China; ^4^ Medical School, Kunming University of Science and Technology, Kunming, China; ^5^ Department of Ophthalmology, Kunming Aier Eye Hospital, Kunming, China

**Keywords:** ferroptosis, diabetic retinopathy, cell death, glutathione peroxidase 4, reactive oxygen species

## Abstract

Ferroptosis is iron-dependent regulatory cell death (RCD). Morphologically, ferroptosis is manifested as mitochondrial atrophy and increased mitochondrial membrane density. Biochemically, ferroptosis is characterized by the depletion of glutathione (GSH), the inactivation of glutathione peroxidase 4 (GPX4), and an increase in lipid peroxides (LPO)and divalent iron ions. Ferroptosis is associated with various diseases, but the relationship with diabetic retinopathy(DR) is less studied. DR is one of the complications of diabetes mellitus and has a severe impact on visual function. The pathology of DR is complex, and the current treatment is unsatisfactory. Therefore, exploring pathogenesis is helpful for the clinical treatment of DR. This paper reviews the pathological mechanism of ferroptosis and DR in recent years and the involvement of ferroptosis in the pathology of DR. In addition, we propose problems that need to be addressed in this research field. It is expected to provide new ideas for treating DR by analyzing the role of ferroptosis in DR.

## 1 Introduction

Cell death is an essential process of life and means the end of cell life. In the past, the Nomenclature Committee on Cell Death (NCCD) has developed guidelines to define and explain cell death from morphological, biochemical, and functional perspectives (1). As the field of cell death continues to evolve and new signaling pathways are discovered, NCCD adjusts how different cell deaths are defined and provides an updated classification of cell death patterns (1). Currently, cell death can be classified into accidental cell death (ACD) and regulatory cell death (RCD) based on functional aspects (2). ACD can be triggered by unexpected attacks and injuries, such as exposure to extreme physicochemical or mechanical stressors, which would overwhelm control mechanisms.

In contrast, RCD is a regulated signaling cascade executed by specific effector molecules and carries unique biochemical, functional, and immunological consequences (3). RCD can be divided into several subroutines based on its molecular characteristics, several of which have clear physiological significance (e.g., necroptosis and pyroptosis). In contrast, others may be cellular responses to specific toxins and do not reflect normal physiology(e.g., ferroptosis, entotic cell death, netotic cell death, parthanatos, lysosome-dependent cell death, autophagy-dependent cell death, alkaliptosis, and oxeiptosis) (3). Ferroptosis is a form of RCD, a concept first proposed by Dixon and colleagues in 2012 (4).

Ferroptosis is cell death that depends on iron metabolism disorder and is characterized by intracellular lipid peroxides (LPO) accumulation. Specific morphological changes in ferroptosis include mitochondrial atrophy and increased mitochondrial membrane density (5). Biochemically, ferroptosis is characterized by the depletion of glutathione (GSH), the decrease of glutathione peroxidase 4 (GPX4) activity, and the accumulation of Fe^2+^ and LPO, thus destroying the typical structure and function of cells (4, 6).

Diabetic retinopathy is one of the significant complications of diabetes mellitus, which can cause visual impairment and blindness. The severity is divided into non-proliferative diabetic retinopathy (NPDR) and proliferative diabetic retinopathy (PDR). The earliest morphological sign of NPDR is the formation of retinal microaneurysms. With the further progress of the diseases, blot hemorrhage, hard exudation, and cotton wool spots in the retina were observed (7). PDR is characterized by neovascularization, fibrovascular proliferation, and leakage of retinal capillaries. Vitreous hemorrhage and tractional retinal detachment develop as the disease progresses (8). The occurrence of DR may be related to the abnormal regulation of many biochemical pathways caused by elevated blood glucose levels. This abnormal regulation ultimately leads to superoxide production and oxidative stress burden in the retinal tissue. Meanwhile, inflammation, neovascularization, and disruption of the blood-retinal barrier are also involved in developing DR (9, 10).

Ferroptosis may be associated with some diseases, such as cancer, brain diseases, immune system diseases, and ischemia/reperfusion. Pathological mechanisms such as oxidative stress, inflammation, and neovascularization are associated with the occurrence and development of these diseases. DR is a metabolic disorder with a similar pathology to these diseases, so DR may also be associated with iron-dependent cell death. However, comprehensive information on the pathophysiology of ferroptosis in DR needs to be comprehensive. In this review, we focus on the recent research development of ferroptosis and its pathological mechanism in DR. Meanwhile, and this paper reviews possible therapeutic targets for ferroptosis in DR. In addition, some questions related to this research field and future research ideas are proposed.

## 2 Mechanisms of ferroptosis

Ferroptosis is a type of RCD with unique morphology and pathological mechanisms. The cystine/Glutamate Antiporter Pathway, System Xc-, is considered to be a critical signaling pathway associated with iron-dependent cell death (5). Lipid peroxidation and iron metabolism are also important events in ferroptosis (11). Meanwhile, GPX4 is a crucial enzyme that degrades peroxides and inhibits ferroptosis. Physiologically, Fe^3+^ enters cells through the transferrin receptor (TFR) and is converted to Fe^2+^, an essential substance for cellular energy metabolism. However, excess Fe^2+^ reduces oxygen to form superoxide radicals and causes lipid peroxidation (12). Therefore, the inactivation of GPX4 and iron metabolism disorders causes intracellular LPO accumulation (13). Furthermore, studies have suggested that p53, a well-known oncogene, is associated with ferroptosis in tumor cells (14).

### 2.1 System Xc-

A cystine/glutamate antiporter, System Xc-, is an amino acid anti-transporter distributed in phospholipid bilayers (15). System Xc- comprises a twelve-channel transmembrane transporter protein SLC7A11 coupled to a single-channel transmembrane regulator protein SLC3A2 via a disulfide bond (16). It imports cystine and exports glutamate, which is critical in maintaining intracellular GSH levels and extracellular cystine/cysteine redox balance (17). The cystine taken up is reduced to cysteine in cells, which is involved in the synthesis of GSH.

### 2.2 GPX4 and GSH

Among the members of the GPX family, GPX4 plays a vital role in ferroptosis, mainly by inhibiting the formation of LPO. GSH acts as an essential cofactor of GPX4, which reduces hydrogen peroxide and organic peroxides to water and corresponding alcohols, thereby eliminating cytotoxicity (11). Therefore, inhibition of GPX4 activity leads to the accumulation of LPO, a critical factor in ferroptosis (18). It was discovered that removing GPX4 in mouse tissues leads to ferroptosis in the corresponding cells (19). Complete deletion of the GPX4 gene has been reported to be lethal in the embryo, resulting in the death of specific cell types in particular tissues (20, 21).

### 2.3 Lipid peroxidation

Both non-enzymatic and enzymatic methods can induce lipid peroxidation, which is crucial in cell ferroptosis (22). Non-enzymatic radical chain reactions are related to Fenton chemistry and produce highly toxic hydroxyl and peroxy radicals. Enzyme-dependent reaction processes involve iron-containing enzymes such as lipoxygenase and arachidonate lipoxygenase (ALOX) (23, 24). Free polyunsaturated fatty acids(PUFAs) are esterified into membrane phospholipids and oxidized as substrates for synthesizing lipid signal transducers (25). PUFA-containing phospholipids (PUFA-PLS) lipid radicals extract protons from adjacent polyunsaturated fatty acids and trigger a new round of lipid oxidation, further spreading oxidative damage from one lipid to another and accelerating the production of LPO (11). Therefore, intracellular lipid peroxidation is regulated by the content and location of PUFA. The exact mechanism by which lipid peroxidation induces ferroptosis and the precise location in cells where this phenomenon occurs are being investigated.

### 2.4 Iron metabolism

As a trace element, iron plays an essential role in maintaining the normal physiological metabolism of the body. Fe^2+^ absorbed by the intestine can be oxidized to Fe^3+^ by ceruloplasmin. Fe^3+^ binds to transferrin(TF) and forms a complex with transferrin receptors 1(TFR1)on the cell membrane before entering the cell (26). Fe^3+^ is reduced to Fe^2+^ in the cell, some are stored in unstable iron pools, and others are bound to ferritin. The iron in ferritin is released by lysosomal degradation, increasing intracellular iron concentration (27). Fe^2+^ reduces oxygen to form superoxide radicals, which induce lipid peroxidation and thus lead to cell ferroptosis (28).

### 2.5 P53

Recent studies have shown that tumor suppressor p53-induced ferroptosis can be a tumor suppressive mechanism. P53 was first reported to induce cell ferroptosis through transcriptional repression of SLC7A11, a specific system-Xc subunit (29). After combining with p53-specific binding proteins, p53 acts on SLC7A11 and inhibits its expression in the promoter region, resulting in enhanced sensitivity of cancer cells to ferroptosis inducers (29). In addition to system Xc-, p53 upregulates its downstream recombinant spermidine/spermine/spermine N1-acetyltransferase 1 (SAT1) to induce ferroptosis (30). In human cancers, wild-type p53 is degraded by the oncogene E3 ubiquitin-protein ligase MDM2. Thus, inhibition of MDM2-dependent proteasomes may play a role in p53-induced ferroptosis, providing novel cancer treatment strategies (27).

With the increased research on iron death, we found that it is involved in various pathophysiological processes in cells. However, ferroptosis involves complex metabolic mechanisms, and a single inhibitor is insufficient evidence (31). It is to be explored how to avoid the interference of other mechanisms in the demonstration of cell ferroptosis. In addition, the specific molecular mechanisms and pathways of ferroptosis in disease remain to be explored.

## 3 Diabetic retinopathy

**Figure 1:**
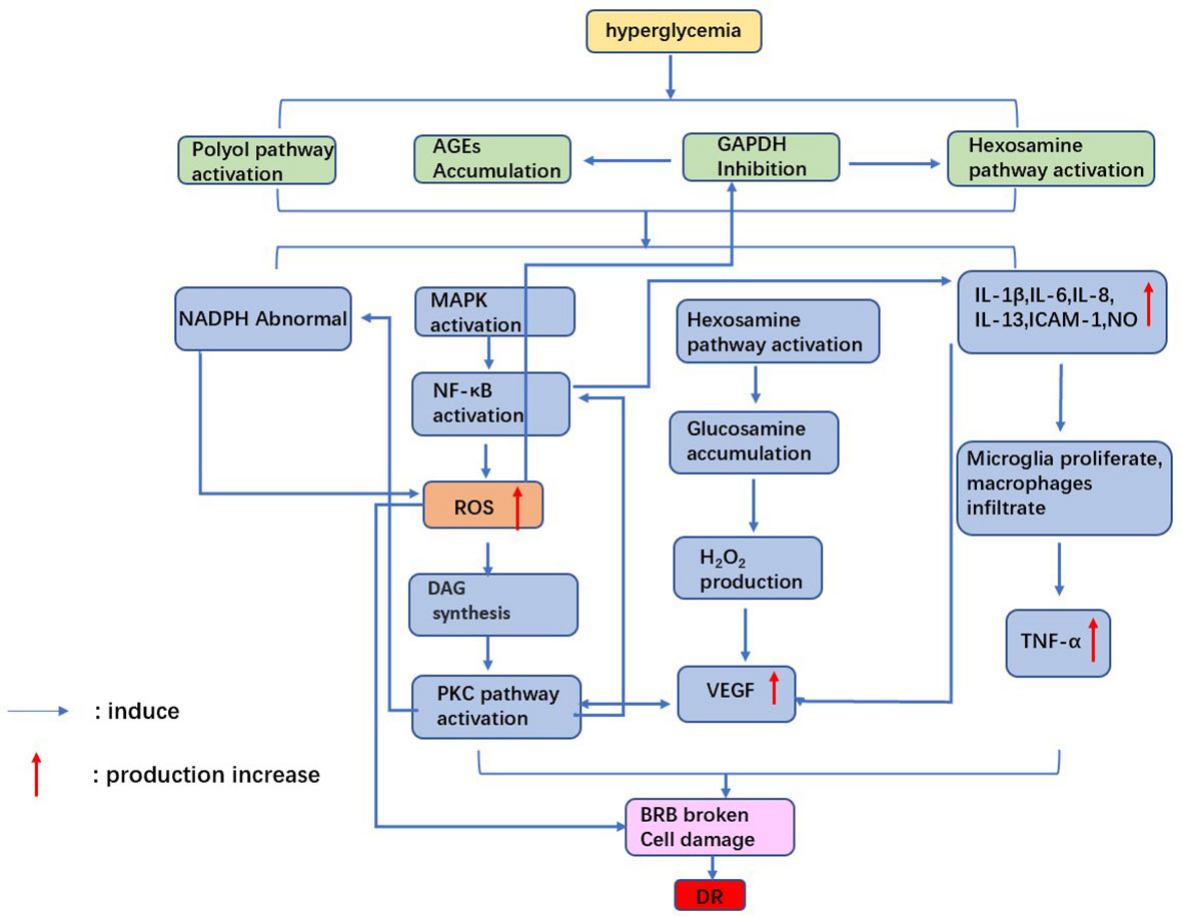
Mechanism of diabetic retinopathy. Hyperglycemia leads to oxidative stress and inflammatory factor release, which damages retinal cells. the accumulation of VEGF causes neovascularization. Ultimately, the blood-retinal barrier is broken, causing diabetic retinopathy.

Although many of the underlying pathogenesis of DR are not fully understood, oxidative stress, inflammation, neovascularization, and disruption of the blood-retinal barrier are involved in the development of DR (7, 8). This review will describe the known pathological mechanisms (**Figure 1**).

### 3.1 Oxidative stress

Oxidative stress generated by hyperglycemia-induced mitochondrial superoxide excess is one of the pathological mechanisms of DR (32). Massive accumulation of ROS leads to mitochondrial dysfunction, apoptosis, local Inflammation, and microvascular dysfunction, significantly affecting all DR pathogenesis (33). Under hyperglycemia, glucose binds to large molecules such as proteins, lipids, and amino acids in nucleic acids, forming AGEs (34). Accumulation of AGEs can cause retinal vascular obstruction, resulting in retinal ischemia and activation of intracellular signaling pathways such as mitogen-activated protein kinase (MAPK) and nuclear transcription factors-κB(NF-κB). These processes trigger cellular hypoxia, ROS production, and cell damage (35). Furthermore, high levels of ROS inhibit glyceraldehyde-3-phosphate dehydrogenase (GAPDH) activity, leading to a flow of glycolytic products into the hexosamine pathway (36). Glucosamine production from activated hexosamine increases hydrogen peroxide production, further leading to cell oxidative damage and enhanced vascular permeability. Meanwhile, inhibition of GAPDH will induce the activity of the AGE pathway, further enhancing oxidative stress in retinal cells (37).

Protein kinase C (PKC) is a serine/threonine kinase involved in signal transduction that responds to the stimulation of specific hormones, neurons, and growth factors (38). ROS accumulation and diacylglycerol (DAG) synthesis under hyperglycemia leads to activation of the PKC pathway. It was found that activation of the PKC pathway could induce endothelial cell damage through increased endothelial cell permeability, altered NO bioavailability, and reduced prostaglandin production (39). In addition to the above pathways, hyperglycemia causes abnormalities in polyol pathways, resulting in reduced NADPH availability and increased cellular sensitivity to oxidative stress (40). On the one hand, retinal cells are exposed to oxidative damage induced by high glucose. On the other hand, retinal antioxidant capacity is reduced under high glucose. Therefore, the combined effect of both factors causes retinal damage.

### 3.2 Inflammation

Chronic Inflammation is associated with developing diabetes and its complications (41, 42). For example, hyperglycemia causes cellular damage by inducing microglia to secrete cytokines and other inflammatory molecules and disrupting the balance of the cellular environment (43). Shelton et al. (44) found that IL-1β, IL-6, IL-8, IL-13, ICAM-1, and NO increased in Muller cells and retinal vascular endothelial cells during DR, which confirmed that inflammatory factors were related to the development of DR.

In addition, increased diacylglycerol (DAG) production via the glycolytic pathway activates PKC under hyperglycemia (45). Activation of PKC drives overexpression of NADPH oxidase and NF-κB in cells, thereby exacerbating DR-related oxidative stress and inflammatory responses (46). It was found that the plasma and vitreous concentrations of TNF-α were increased in patients with diabetes, and the plasma TNF-α concentration was related to the severity of DR (47).In diabetic rats, the vitreous TNF-α concentration was related to the increased permeability of BRB (48). Therefore, the chronic inflammatory response caused by hyperglycemia creates long-term damage to retinal cells, affecting the repair ability of cells and promoting DR development.

### 3.3 Vascular endothelial growth factor and neovascularization

Neovascularization is one of the critical features of PDR. Occlusion of retinal microvessels can lead to retinal ischemia and neovascularization. This process is accompanied by the release of vascular endothelial growth factor (VEGF), a glycoprotein that increases retinal vascular permeability and promotes neovascularization. There are four subtypes of VEGF, including VEGF-121, -165, -189, and -206. VEGF-165 has proven to be most closely associated with DR pathology (49).

VEGF destroys retinal cells mainly through the following factors. Firstly, VEGF induces ICAM-1 expression and leukocyte adhesion in the retina, resulting in BRB rupture, capillary nonperfusion, and endothelial cell injury. Secondly, VEGF promotes tight junction disassembly, vascular permeability, and BRB disruption by inducing PKC activation and phosphorylation of tight junction proteins (50). Activation of PKC-B2 isoforms is reported to be critical in the mechanism of VEGF-induced DR neovascularization (51). Finally, VEGF induces the expression of serine proteinases, tissue fibrinogen activators, and metalloproteinases while significantly reducing tissue inhibitors of metalloproteinases. These factors lead to abnormal retinal angiogenesis and proliferation (52).

### 3.4 Blood-retinal barrier impairment

The outer blood-retinal barrier(oBRB) comprises the choroid, Bruch’s membrane, and RPE. The inner blood-retinal barrier (iBRB) comprises the retinal endothelium and pericytes, essential for maintaining retinal tissue integrity and normal retinal function (53). One of the causes of retinal edema and macular edema in diabetic patients is BRB disruption and fluid accumulation in the retinal space. Injury of BRB under hyperglycemia may be related to the following factors.

On the one hand, Inflammation caused by hyperglycemia plays an essential role in destroying retinal integrity. It was found that IL-6 levels in retinal tissue were elevated in an animal model of diabetes, which would upregulate levels of mRNA encoding microglia chemokines and result in microglia proliferation (54). TNF-α production and secretion by IL-6-treated microglia reduced zonula occludens-1(ZO-1) in RPE cells and disrupted the tight junction complex, thereby affecting the integrity of the oBRB. In addition, IL-1β and IL-8 also upregulated the expression of VEGF in diabetic animal models, which resulted in a substantial reduction in pericytes, capillary deterioration, and breakdown of BRB.

On the other hand, the integrity of RPE cells is also closely related to the electrical activity of the cell membrane. Na+/k+-ATPase is an essential source of energy for RPE cells. It uses the energy of ATP to pump 3 Na+ out of the cell and every 2 K+ into it. Na+/k+ - ATPase activity is significantly reduced in a hyperglycemic environment, increasing intracellular osmotic pressure, retinal cell edema, and retinal barrier (55).

Retinal tissue comprises multiple cells, so diabetic retinopathy involves pathological changes in different cells, which is complicated and elongated. Based on the continuous development of clinicopathological and experimental data over the past years, a broader view of diabetic retinopathy and its progress is now available (32). However, treating diabetic retinopathy in clinical practice mainly focuses on the intermediate and late stages. More in-depth studies on the pathogenesis are still needed for early treatment and prevention.

## 4 Research progress into ferroptosis in diabetic retinopathy

### 4.1 Reactive oxygen species

Oxidative stress is associated with developing several neuroretinal degenerative diseases, such as AMD and DR. Oxidative stress is cell damage caused by an imbalance between oxidants and antioxidants. It causes excessive accumulation of ROS and damage to cellular proteins, lipids, and DNA. On the one hand, hyperglycemia disrupts mitochondrial homeostasis to produce massive amounts of ROS, causing local tissue and cellular hypoxia. Long-term chronic hypoxia, in turn, aggravates oxidative stress damage to cells. Dimethyloxallyl Glycine (DMOG) stabilizes hypoxia-inducible factors and promotes cellular adaptation to hypoxia (56). Sodium iodate (SIO) and DMOG-treated ARPE-19 increase superoxide dismutase activity and Fe^3+^ levels, intensifying the Fenton reaction (56).

On the other hand, ROS reacts with phospholipids in biological membranes, enzymes, and side chains of polyunsaturated fatty acids and nucleic acids to form LPO such as malonaldehyde (MDA) and 4-hydroxynonenal (HNE) through lipid peroxidation. Eventually, the fluidity and permeability of cell membranes change, and cell structure and function are affected. Zhou Jing et al. discovered that the ROS scavenger, n-acetyl-l-cysteine (NAC), inhibited high glucose-induced ferroptosis in RPE cells (ARPE-19) after the elimination of ROS (45). Therefore, oxidative stress was shown to be involved in cell ferroptosis under hyperglycemia.

As an antioxidant, nuclear factor erythroid 2-related factor 2 (NRF2) is a redox-sensitive essential leucine zipper region transcription factor, which plays a crucial role in protecting the retinal vascular system from ROS-related damage (35). Nrf2 enters the nucleus by binding to genes associated with multiple antioxidant reaction elements, thereby promoting the expression of target genes such as GPX, heme oxidase-1 (HO-1), and glutamine-cysteine ligase catalytic subunit (GCLC) (57). The activity of NRF2-related antioxidant enzymes decreased in an *in vitro* model of diabetes. Therefore, NRF2 depletion leads to uninhibited NOX2 activation and triggers ROS accumulation (58), which causes cell damage in diabetes models. Several studies have identified some factors associated with the regulation of Nrf2. For example, Silencing information regulator 1(Sirt1), a nicotinamide adenine nucleotide (NAD) -dependent protein deacetylase, has been shown to regulate Nrf2 expression and activity positively, thereby inhibiting cell ferroptosis (59). Ras-selection-lethal compound 3(RSL3) inhibits the activity of GPX4, which leads to the accumulation of ROS and reduces the antioxidant capacity of cells (13). However, intraperitoneal injection of RSL3 increased Nrf2 expression in mouse microglia, thus partially inhibiting the production of inflammatory cytokines and, to some extent increasing the resistance of cells to ferroptosis (60).

**Figure 2:**
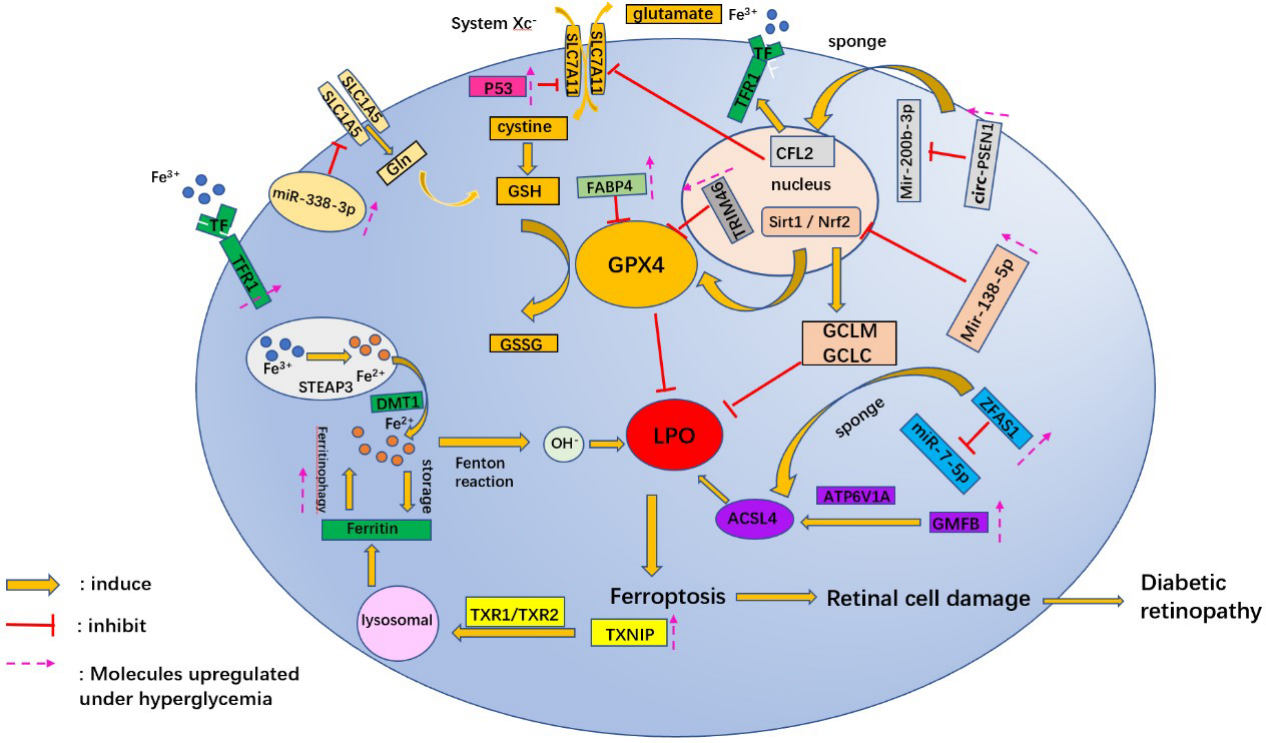
A schematic picture shows the potential mechanism associated with ferroptosis in diabetic retinopathy. Gln, glutamine; GSH, L-Glutathione; GPX4, glutathione peroxidase 4; GSSG, Oxidized glutathione; TRIM46, tripartite motif 46; CFL2, Cofilin2; Mir, micro RNA; circ-PSEN1, circular RNA- Presenilin 1; sirt1, Sirtuins 1; Nrf2, nuclear factor-erythroid 2-related factor; GCLM, glutamate-cysteine ligase modifier subunit; GCLC, glutamate-cysteine ligase catalytic subunit; FABP4, fatty acid binding protein 4; LPO, lipid peroxides; GMFB, glial maturation factor B; ATP6V1A, 1V-type proton ATPase catalytic subunit A; ACSL4, acyl-CoA synthetase long-chain family member 4; TF, transferrin; TFR, transferrin receptor; STEAP3, STEAP family member 3; DMT1, Divalent metal cation transporter 1; TXNIP, thioredoxin-interacting protein; TrxR1, thioredoxin reductase 1; ZFAS1, zinc finger antisense 1.

Recent studies have also identified target miRNAs associated with oxidative stress in DR (61), such as miR-338-3p (62). miR-338-3p targets the 3 ‘untranslated region (3’UTR) of SLC1A5 for inhibition and degradation. Hyperglycemia triggers ferroptosis in ARPE-19 cells by regulating the Mir-338-3p/SLC1A5 axis. The main mechanism is that overexpression of Mir-338-3p and inhibition of SLC1A5 promote intracellular ROS, total iron, and ferrous iron levels (45) (**Figure 2**).

### 4.2 GPX4 and GSH

GPX protects cells from oxidative stress damage and inhibits cell ferroptosis. The outer segments of photoreceptors are the site of high concentrations of PUFAs and are vulnerable to oxidative stress (63). Knockdown of GPX4 isoforms completely in mouse photoreceptors leads to an early degenerative phenotype, which reveals the importance of GPX4 for photoreceptor survival (21). Cultivation of RPE cells in a GSH-depleted medium resulted in decreased GPX4 expression and increased LPO production. In addition, GSH depletion induced premature senescence in RPE cells, as evidenced by an increased percentage of senescence-associated β-galactosidase-positive cells, increased senescence-associated heterochromatin foci, and cell cycle arrest at the G1 phase. Therefore, GSH depletion leads to RPE cell degeneration and ferroptosis (64).

miRNAs regulate post-transcriptional gene expression and cell ferroptosis by affecting GPX4 expression in hyperglycemic conditions. Overexpression of Mir-138-5p inhibited the activity of the Sirt1/Nrf2 pathway in a hyperglycemic environment and reduced the antioxidant capacity of RPE cells. The primary mechanism was to inhibit downstream molecules of Sirt1/Nrf2, such as GPX4, glutamine-cysteine ligase modified subunit (GCLM), and glutamine-cysteine ligase catalytic subunit (GCLC) proteins (65). Tang X et al. (66) discovered that Astragaloside IV (AS-IV) attenuated ferroptosis in RPE cells by inhibiting Mir-138-5p expression. In addition to miRNA, lncRNAs are also involved in regulating gene expression of ferroptosis in retinal cells. lncRNA zinc finger antisense 1 (ZFAS1) expression was upregulated in hRECs cultured with high glucose. ZFAS1 may promote the expression of downstream mRNA ACSL4 and reduce the level of GPX4 by competitive binding to miR-7-5p under a high glucose environment (66).

High glucose levels have been found to promote the activation of the oncogene p53 (67). To further determine whether the p53-System Xc–GSH axis is involved in HG- and IL-1β-induced iron death in endothelial cells, Luo EF et al. transiently transfected HUVECs with p53 siRNA or NC siRNA, followed by treatment with NG, HG, and IL-1β. The results revealed that p53 siRNA significantly reduced the expression of HG- and IL-1β-induced xCT and GSH (68).

Ferrostatin-1 was the first ferroptosis inhibitor developed to inhibit LPO by scavenging the initiating alkoxy radical and other rearrangement products (69). It was found that Ferrostatin 1 significantly reduced the expression of MDA, one of the lipid peroxidation products, as well as increased the proteins of SLC7A11 and GPX4 in photoreceptor cells (54). Besides), Inhibitors (e.g., elastin (4), sorafenib (70), or sulfasalazine (71))that act on SLC7A11 lead to depletion of GSH and inactivation of GPX4, resulting in LPO accumulation and cell ferroptosis.

### 4.3 Lipid peroxidation

As is known, one of the critical features of ferroptosis is the accumulation of LPO. High glucose leads to the destruction of the blood-brain barrier and the release of many divalent iron ions, which causes the overproduction of ROS in the Fenton reaction and oxidizes cell membrane lipids (23). FABP4 is a small molecule protein vital in maintaining glucose and lipid homeostasis. FABP4 is highly expressed in diabetic complications. Inhibition of FABP4 ameliorates high glucose-induced glomerular apoptosis and increases serum insulin concentration, thereby attenuating the progression of diabetes-relevant diseases. Therefore, FABP4 participates in and regulates the development of diabetes-related diseases (72). Fan X et al. used the FABP4 inhibitor BMS309403 to enhance the activity of GPX4 and reduce lipid peroxidation, thereby protecting diabetic mouse retinal cells from oxidative stress (73).

Other molecules, such as TRIM46, are also involved in regulating lipid Peroxidation in hyperglycemia. TRIM46, a gene localized on chromosome 1q21, could mediate the ubiquitination of GPX4. Overexpression of TRIM46 in high-glucose treated human retinal microvascular endothelial cells inhibits GPX4 activity and increases ROS and MDA, whereas TRIM46 knockdown produces the opposite effect (74). Furthermore, TRIM46 overexpression aggravates the damage of Human retinal microvascular endothelial cells (HRCECs) in high glucose conditions by promoting the degradation of IκBα and the activation of the NF-κB pathway (75).

In addition, circRNAs affect cell ferroptosis in DR by regulating the expression of miRNAs (76). For example, the knockdown of circRNA PSEN1 ameliorates high glucose-induced ferroptosis in RPE cells via the Mir-200b-3p/cofilin-2(CFL2)axis. MiR-200b-3p is sponged by circ-PSEN1, which leads to enhanced expression of CFL2. Overexpression of CFL2 results in repression of mRNA and protein expression of GPX4 and SLC7A11, while expression of TFR1 is promoted (77). These factors cause an increase in intracellular Fe^2+^ and LPO, which leads to cell ferroptosis.

### 4.4 Mitochondrial and lysosomal dysfunction

*Mitochondria* are organelles that provide energy for various physiological activities of the cell. Lysosomes are rich in hydrolytic enzymes and perform intracellular digestive functions. Mitochondrial and lysosomal dysfunction can induce cell ferroptosis. Mitochondria consume large amounts of glucose and oxygen to produce ATP. ATP is necessary to satisfy the high energy demand required for retinal (78) so a significant reduction in ATP levels can affect visual function. However, many reactive oxygen radicals accompanying ATP production can damage mitochondrial membranes, proteins, and mitochondrial DNA (79). ROS destroy mitochondria in a high glucose environment, and damaged mitochondria produce less ATP but continue to produce ROS (80). Under hyperglycemia, thioredoxin-interacting protein (TXNIP) expression is enhanced in ARPE-19 cells (81). It induces autophagy flux, mitochondrial dysfunction, and lysosomal instability by binding to and inhibiting thioredoxin reductases 1 and 2 (TrxR1 and TrxR2) (82). A large amount of Fe^2+^ is released from the lysosome during this process. The released Fe^2+^ reacts with hydrogen peroxide to produce many hydroxyl radicals, resulting in lipid peroxidation of lysosomes, mitochondria, and plasma membranes. This process damages the organelles and plasma membrane of the cells (83). Several Dihydrolipoic acids (ALA) derivatives, including Dihydrolipoic acid, are known to protect mitochondria from oxidative stress-induced damage by upregulating the anti-inflammatory and antioxidant defense systems of cells (84).

RPE cells phagocytose the exfoliated outer segment membrane discs of photoreceptors to provide nutrients to the retinal neuroepithelial layer. High glucose causes mitochondrial damage, lysosomal overload, and reduced protein digestion ability in RPE cells. Undigested lipofuscin is deposited in the Bruch membrane, thereby damaging the integrity of the RPE barrier function. Therefore, removing damaged mitochondria by mitochondrial phagocytosis reduces intracellular ROS and protects cells from oxidative stress damage. Thangal Yumnamcha et al. (82) reduced the incidence of ferroptosis in RPE cells by combining SS31 (mitochondrial antioxidant), ammonia oxidant (inhibition of TBK1 and normalization of mitochondrial phagocytic flux), and tranilast (inhibition of NLRP3) to inhibit mitochondrial dysfunction. Thus, the three-drug combination may be helpful in the treatment of mitochondria-associated neurodegenerative diseases, such as DR.

In hyperglycemia, the function of lysosomes is affected by several acidic proteins. It was found that the concentration of glial maturation factor-β (GMFB) in the vitreous cavity of diabetic rats was significantly increased (65). GMFB affects the degradation process of autophagolysosome through ATP6V1A translocation and induces the accumulation of ACSL4 (partner-mediated autophagy substrate), ultimately affecting normal cell metabolism and destroying the physiological functions of the retina (65).

## 5 Conclusion and outlook

Ferroptosis has received more research and attention as a mode of RCD. This paper provides a more comprehensive description of the molecules and pathways associated with iron toxicity. It mainly includes abnormal regulation of System Xc-, depletion of GSH, and inactivation of GPX4, as well as abnormal intracellular iron metabolism, accumulation of LPO, and regulation of P53. The molecules and pathways associated with ferroptosis are complex and require further investigation. There are few studies on the relationship between ferroptosis and ocular diseases. There is no comprehensive review of ferroptosis in the pathogenesis of DR. Therefore, in this paper, the existing mechanisms of ferroptosis associated with the development of DR are described to understand the pathology of DR further (**Tables 1**, **2**).

**Table 1:** Mediators of ferroptosis in diabetic retinopathy.

	Mediators	Models	Mechanisms	References
Inducers	RSL3	*in vitro* (human retinal capillary endothelial cells)	inactivating GPX4, Reversing the protective effect of TRIM46 transactivation	(55)
	GW9662	*In vivo* (STZ mice)	Promoting the expression of SLC7A11 and GPX4	(85)
Inhibitors	Astragaloside-IV (AS-IV)	*in vitro* (ARPE-19 cells)	increasing mir-138-5p, Sirt1 and Nrf2 expression	(86)
	GMFB antibody	*In vivo* (STZ rats)	reducing ACSL4 and MDA in the retina, reducing autophagosomes	(63)
	Lysosome activator NKH477			
	CMA activator QX77			
	SS31	*in vitro* (ARPE-19 cells)	mitochondrial antioxidant	(62)
	Amlexanox		inhibiting inflammatory kinases TBK1 and normalizing mitophagy flux	
	tranilast		inhibiting pro-inflammatory caspase-1(NLRP3), Improving mitochondrial dysfunction	
	ferrostatin-1	*in vitro* (ARPE-19 cells)	decreasing ROS, mitochondrial superoxide, total iron and Fe^2+^	(43)
	N-acetyl-L-Cysteine (NAC)			
	GW9662	*In vivo* (STZ mice)	Promoting the expression of SLC7A11 and GPX4	(85)

**Table 2:** Pathogenesis of ferroptosis in diabetic retinopathy.

pathological characteristics	mechanism	Cell/animal	Inducers/Inhibitors	Reference
Oxidative stress	ROS, MDA and HNE express enhance	ARPE-19	ROS scavenger, NAC inhibitor	(45, 56)
	NRF2 suppression, NOX2activation, and ROS accumulation	HRCEC	RSL3 suppression	(13, 58, 59)
	miR-338-3p over-express, SLC1A5 suppression, ROS accumulation	ARPE-19	miR-338-3p inhibitors, SLC1A5 overexpression vectors	(45, 61)
GPX4 and GSH	Mir-138-5p overexpression, Sirt1-Nrf2 suppression, GPX4, GCLM and GCLC decrease	ARPE-19	AS-IV	(66, 85)
	ZFAS1 and ACSL4 express enhance, GPX4 suppression	hRECs, C57BL mice	–	(87)
Lipid Peroxidation	ABP4 express enhance, GPX4 suppression, LPO accumulation	C57BL mice, ARPE-19	BMS309403,(Erastin inducer) GW9662 (PPARγ inhabitor)	(72, 73)
	TRIM46 over-express, IκBαdegradation, NF-κB pass way activation, GPX4 suppression, LPO accumulation	HRCECs	TRIM46 knockout	(74, 75)
	circ-PSEN1 up-regulation, CFL2 and TFR1 express enhance	ARPE-19	si-circ-PSEN1	(76, 77)
Mitochondrial and Lysosomal Dysfunction	TXNIP express enhance, Induction of autophagic flux, mitochondrial dysfunction and lysosomal instability	ARPE-19	ALA derivative, SS31(Mitochondrial antioxidant), Ammonia oxidizer, Trinestrol	(81–84)
	GMFB express enhance, ATP6V1Atranslocation, ACSL4 accumulation	ARPE-19	CMA activator QX77, ferroptosis inhibitor-1	(65)

DR is a metabolism-related disease with complex pathology that seriously threatens visual function. Current treatment for DR includes control of blood glucose and blood pressure, retinal laser photocoagulation, anti-VEGF, intravitreal corticosteroids, and vitrectomy. Photocoagulation causes some damage to retinal tissue, and vitreous corticosteroids may lead to increased intraocular pressure and secondary glaucoma. Multiple vitreous injections increase the risk of intraocular infection. Moreover, vitrectomy may cause medical damage to the retina, and surgical treatment of the cause of DR cannot be performed. Therefore, more than the effects of current treatments is needed. This paper discusses the role of ferroptosis in the development of DR and complements the pathogenesis of DR, providing new ideas for treating DR.

However, current research focuses on animal and *in vitro* experiments. Although the relevance of ferroptosis to DR has been confirmed, whether its pathological mechanisms in animal models or *in vitro* are closely aligned with those in humans needs further proof. It is also unclear whether ferroptosis inhibitors suppress the development of DR in humans. Therefore, the clinical application of ferroptosis-related therapy still needs further exploration. Future research on ferroptosis will have positive significance for DR prevention, control, and treatment.

## Author contributions

WH completed the manuscript. LC assisted in the completion of the manuscript. YM was responsible in the revision of articles XL assisted in the literature search for the manuscript. All authors contributed to the article and approved the submitted version.

## References

[1] Galluzzi L Vitale I Aaronson SA Abrams JM Adam D Agostinis P. Molecular mechanisms of cell death: recommendations of the nomenclature committee on cell death 2018. Cell Death Differ (2018) 25(3):486–541. doi: 10.1038/s41418-017-0012-4 29362479 PMC5864239

[2] Galluzzi L Bravo-San Pedro JM Vitale I Aaronson SA Abrams JM Adam D. Essential versus accessory aspects of cell death: recommendations of the NCCD 2015. Cell Death Differ (2015) 22(1):58–73. doi: 10.1038/cdd.2014.137 25236395 PMC4262782

[3] Tang D Kang R Berghe TV Vandenabeele P Kroemer G. The molecular machinery of regulated cell death. Cell Res (2019) 29(5):347–64. doi: 10.1038/s41422-019-0164-5 PMC6796845 30948788

[4] Dixon SJ Lemberg KM Lamprecht MR Skouta R Zaitsev EM Gleason CE. Ferroptosis: an iron-dependent form of nonapoptotic cell death. Cell (2012) 149(5):1060–72. doi: 10.1016/j.cell.2012.03.042 PMC3367386 22632970

[5] Ke B Tian M Li J Liu B He G. Targeting programmed cell death using small-molecule compounds to improve potential cancer therapy: ANTICANCER COMPOUNDS TARGETING CELL DEATH. Med Res Rev (2016) 36(6):983–1035. doi: 10.1002/med.21398 27357603

[6] Friedmann Angeli JP Schneider M Proneth B Tyurina YY Tyurin VA Hammond VJ. Inactivation of the ferroptosis regulator Gpx4 triggers acute renal failure in mice. Nat Cell Biol (2014) 16(12):1180–91. doi: 10.1038/ncb3064 PMC4894846 25402683

[7] Kollias AN Ulbig MW. Diabetic retinopathy. Deutsches Ärzteblatt international (2010) 107(5):75–83. doi: 10.3238/arztebl.2010.0075 PMC2828250 20186318

[8] Tan Y Fukutomi A Sun MT Durkin S Gilhotra J Chan WO. Anti-VEGF crunch syndrome in proliferative diabetic retinopathy: a review. Survey Ophthalmol (2021) 66(6):926–32. doi: 10.1016/j.survophthal.2021.03.001 33705807

[9] Joussen AM Smyth N Niessen C. Pathophysiology of diabetic macular edema. (2007) 39:1–12. doi: 10.1159/000098495 17245075

[10] Bandello F Lattanzio R Zucchiatti I Del Turco C. Pathophysiology and treatment of diabetic retinopathy. Acta Diabetol (2013) 50(1):1–20. doi: 10.1007/s00592-012-0449-3 23277338

[11] Su Y Zhao B Zhou L Zhang Z Shen Y Lv H. Ferroptosis, a novel pharmacological mechanism of anti-cancer drugs. Cancer Letters (2020) 483:127–36. doi: 10.1016/j.canlet.2020.02.015 32067993

[12] Gkouvatsos K Papanikolaou G Pantopoulos K. Regulation of iron transport and the role of transferrin. Biochim Biophys Acta (BBA) - Gen Subjects (2012) 1820(3):188–202. doi: 10.1016/j.bbagen.2011.10.013 22085723

[13] Yang WS. Regulation of ferroptotic cancer cell death by GPX4. Cell (2014) 156(1-2):317–31. doi: 10.1016/j.cell.2013.12.010 PMC4076414 24439385

[14] Wang SJ Li D Ou Y Jiang L Chen Y Zhao Y. Acetylation is crucial for p53-mediated ferroptosis and tumor suppression. Cell Rep (2016) 17(2):366–73. doi: 10.1016/j.celrep.2016.09.022 PMC5227654 27705786

[15] Li J Cao F Yin HL Huang ZJ Lin ZT Mao N. Ferroptosis: past, present and future. Cell Death Dis (2020) 11(2):88. doi: 10.1038/s41419-020-2298-2 32015325 PMC6997353

[16] Sato H Tamba M Ishii T Bannai S. Cloning and expression of a plasma membrane Cystine/Glutamate exchange transporter composed of two distinct proteins. J Biol Chem (1999) 274(17):11455–8. doi: 10.1074/jbc.274.17.11455 10206947

[17] Bridges RJ Natale NR Patel SA. System xc- cystine/glutamate antiporter: an update on molecular pharmacology and roles within the CNS: system xc- cystine/glutamate antiporter. Br J Pharmacol (2012) 165(1):20–34. doi: 10.1111/j.1476-5381.2011.01480.x 21564084 PMC3252963

[18] Yang WS Kim KJ Gaschler MM Patel M Shchepinov MS Stockwell BR. Peroxidation of polyunsaturated fatty acids by lipoxygenases drives ferroptosis. Proc Natl Acad Sci USA (2016) 113(34):E4966–75. doi: 10.1073/pnas.1603244113 PMC5003261 27506793

[19] Tang D Kroemer G. Ferroptosis. Curr Biol (2020) 30(21):R1292–7. doi: 10.1016/j.cub.2020.09.068 33142092

[20] Imai H Hirao F Sakamoto T Sekine K Mizukura Y Saito M. Early embryonic lethality caused by targeted disruption of the mouse PHGPx gene. Biochem Biophys Res Commun (2003) 305(2):278–86. doi: 10.1016/S0006-291X(03)00734-4 12745070

[21] Ueta T Inoue T Furukawa T Tamaki Y Nakagawa Y Imai H. Glutathione peroxidase 4 is required for maturation of photoreceptor cells. J Biol Chem (2012) 287(10):7675–82. doi: 10.1074/jbc.M111.335174 PMC3293550 22207760

[22] Latunde-Dada GO. Ferroptosis: role of lipid peroxidation, iron and ferritinophagy. Biochim Biophys Acta (BBA) - Gen Subjects (2017) 1861(8):1893–900. doi: 10.1016/j.bbagen.2017.05.019 28552631

[23] Stockwell BR Friedmann Angeli JP Bayir H Bush AI Conrad M Dixon SJ. Ferroptosis: a regulated cell death nexus linking metabolism, redox biology, and disease. Cell (2017) 171(2):273–85. doi: 10.1016/j.cell.2017.09.021 PMC5685180 28985560

[24] Feng H Stockwell BR. Unsolved mysteries: how does lipid peroxidation cause ferroptosis? PloS Biol (2018) 16(5):e2006203. doi: 10.1371/journal.pbio.2006203 29795546 PMC5991413

[25] Yang WS Stockwell BR. Ferroptosis: death by lipid peroxidation. Trends Cell Biol (2016) 26(3):165–76. doi: 10.1016/j.tcb.2015.10.014 PMC4764384 26653790

[26] Frazer DM Anderson GJ. The regulation of iron transport: the regulation of iron transport. BioFactors (2014) 40(2):206–14. doi: 10.1002/biof.1148 24132807

[27] Asano T Komatsu M Yamaguchi-Iwai Y Ishikawa F Mizushima N Iwai K. Distinct mechanisms of ferritin delivery to lysosomes in iron-depleted and iron-replete cells. Mol Cell Biol (2011) 31(10):2040–52. doi: 10.1128/MCB.01437-10 PMC3133360 21444722

[28] Ng SW Norwitz SG Norwitz ER. The impact of iron overload and ferroptosis on reproductive disorders in humans: implications for preeclampsia. IJMS (2019) 20(13):3283. doi: 10.3390/ijms20133283 31277367 PMC6651445

[29] Jiang L Kon N Li T Wang SJ Su T Hibshoosh H. Ferroptosis as a p53-mediated activity during tumour suppression. Nature (2015) 520(7545):57–62. doi: 10.1038/nature14344 25799988 PMC4455927

[30] Peng JJ Song WT Yao F Zhang X Peng J Luo XJ. Involvement of regulated necrosis in blinding diseases: focus on necroptosis and ferroptosis. Exp Eye Res (2020) 191:107922. doi: 10.1016/j.exer.2020.107922 31923413

[31] Jiang X Stockwell BR Conrad M. Ferroptosis: mechanisms, biology and role in disease. Nat Rev Mol Cell Biol (2021) 22(4):266–82. doi: 10.1038/s41580-020-00324-8 PMC8142022 33495651

[32] Lechner J O’Leary OE Stitt AW. The pathology associated with diabetic retinopathy. Vision Res (2017) 139:7–14. doi: 10.1016/j.visres.2017.04.003 28412095

[33] Wu MY Yiang GT Lai TT Li CJ. The oxidative stress and mitochondrial dysfunction during the pathogenesis of diabetic retinopathy. Oxid Med Cell Longevity (2018) 2018:1–12. doi: 10.1155/2018/3420187 PMC6145164 30254714

[34] Ott C Jacobs K Haucke E Navarrete Santos A Grune T Simm A. Role of advanced glycation end products in cellular signaling. Redox Biol (2014) 2:411–29. doi: 10.1016/j.redox.2013.12.016 PMC3949097 24624331

[35] Rodríguez ML Pérez S Mena-Mollá S Desco MC Ortega ÁL. Oxidative stress and microvascular alterations in diabetic retinopathy: future therapies. Oxid Med Cell Longevity (2019) 2019:1–18. doi: 10.1155/2019/4940825 PMC6878793 31814880

[36] Du XL Edelstein D Rossetti L Fantus IG Goldberg H Ziyadeh F. Hyperglycemia-induced mitochondrial superoxide overproduction activates the hexosamine pathway and induces plasminogen activator inhibitor-1 expression by increasing Sp1 glycosylation. Proc Natl Acad Sci USA (2000) 97(22):12222–6. doi: 10.1073/pnas.97.22.12222 PMC17322 11050244

[37] Brownlee M. The pathobiology of diabetic complications. Diabetes (2005) 54(6):1615–25. doi: 10.2337/diabetes.54.6.1615 15919781

[38] Kang Q Yang C. Oxidative stress and diabetic retinopathy: molecular mechanisms, pathogenetic role and therapeutic implications. Redox Biol (2020) 37:101799. doi: 10.1016/j.redox.2020.101799 33248932 PMC7767789

[39] Amadio M Bucolo C Leggio GM Drago F Govoni S Pascale A. The PKCβ/HuR/VEGF pathway in diabetic retinopathy. Biochem Pharmacol (2010) 80(8):1230–7. doi: 10.1016/j.bcp.2010.06.033 20599775

[40] Whitehead M Wickremasinghe S Osborne A Van Wijngaarden P Martin KR. Diabetic retinopathy: a complex pathophysiology requiring novel therapeutic strategies. Expert Opin Biol Ther (2018) 18(12):1257–70. doi: 10.1080/14712598.2018.1545836 PMC6299358 30408422

[41] Pollack RM Donath MY LeRoith D Leibowitz G. Anti-inflammatory agents in the treatment of diabetes and its vascular complications. Diabetes Care (2016) 39(Supplement_2):S244–52. doi: 10.2337/dcS15-3015 27440839

[42] Taurone S Spoletini M Ralli M Gobbi P Artico M Imre L. Ocular mucous membrane pemphigoid: a review. Immunol Res (2019) 67(2–3):280–9. doi: 10.1007/s12026-019-09087-7 31327149

[43] Taurone S Ralli M Nebbioso M Greco A Artico M. Attanasio G, et al. The role of inflammation in diabetic retinopathy: a review. European Review for Medical and Pharmacological Sciences (2020) 24(20):10319–29. doi: 10.26355/eurrev_202010_23379 33155187

[44] Shelton MD Distler AM Kern TS Mieyal JJ. Glutaredoxin regulates autocrine and paracrine proinflammatory responses in retinal glial (Müller) cells. J Biol Chem (2009) 284(8):4760–6. doi: 10.1074/jbc.M805464200 PMC2643491 19074435

[45] Zhou J Sun C Dong X Wang H. A novel miR-338-3p/SLC1A5 axis reprograms retinal pigment epithelium to increases its resistance to high glucose-induced cell ferroptosis. J Mol Histol (2022) 53(3):561–71. doi: 10.1007/s10735-022-10070-0 35320491

[46] Koya D King GL. Protein kinase c activation and the development of diabetic complications. Diabetes (1998) 47(6):859–66. doi: 10.2337/diabetes.47.6.859 9604860

[47] Bolinger M Antonetti D. Moving past anti-VEGF: novel therapies for treating diabetic retinopathy. IJMS (2016) 17(9):1498. doi: 10.3390/ijms17091498 27618014 PMC5037775

[48] Bamforth SD Lightman S Greenwood J. The effect of TNF-α and IL-6 on the permeability of the rat blood-retinal barrier *in vivo* . Acta Neuropathol (1996) 91(6):624–32. doi: 10.1007/s004010050476 8781662

[49] Régnier S Malcolm W Allen F Wright J Bezlyak V. Efficacy of anti-VEGF and laser photocoagulation in the treatment of visual impairment due to diabetic macular edema: a systematic review and network meta-analysis. PloS One (2014) 9(7):e102309. doi: 10.1371/journal.pone.0102309 25029255 PMC4100770

[50] Murakami T Frey T Lin C Antonetti DA. Protein kinase Cβ phosphorylates occludin regulating tight junction trafficking in vascular endothelial growth factor–induced permeability *In vivo* . Diabetes (2012) 61(6):1573–83. doi: 10.2337/db11-1367 PMC3357276 22438576

[51] Suzuma K Takahara N Suzuma I Isshiki K Ueki K Leitges M. Characterization of protein kinase c β isoform’s action on retinoblastoma protein phosphorylation, vascular endothelial growth factor-induced endothelial cell proliferation, and retinal neovascularization. Cell Biol (2002) 99(2):721–6. 10.1073/pnas.022644499 PMC117372 11805327

[52] Kaštelan S Orešković I Bišćan F Kaštelan H Gverović Antunica A. Inflammatory and angiogenic biomarkers in diabetic retinopathy. Biochem Med (Online) (2020) 30(3):385–99. doi: 10.11613/BM.2020.030502 PMC7394255 32774120

[53] Young RW Bok D. Participation of the retinal pigment epithelium in the rod outer segment renewal process. J Cell Biol (1969) 42(2):392–403. doi: 10.1083/jcb.42.2.392 5792328 PMC2107669

[54] Tang W Guo J Liu W Ma J Xu G. Ferrostatin-1 attenuates ferroptosis and protects the retina against light-induced retinal degeneration. Biochem Biophys Res Commun (2021) 548:27–34. doi: 10.1016/j.bbrc.2021.02.055 33631670

[55] Xia T Rizzolo LJ. Effects of diabetic retinopathy on the barrier functions of the retinal pigment epithelium. Vision Res (2017) 139:72–81. doi: 10.1016/j.visres.2017.02.006 28347688

[56] Henning Y Blind US Larafa S Matschke J Fandrey J. Hypoxia aggravates ferroptosis in RPE cells by promoting the fenton reaction. Cell Death Dis (2022) 13(7):662. doi: 10.1038/s41419-022-05121-z 35906211 PMC9338085

[57] Ma Q. Role of Nrf2 in oxidative stress and toxicity. Annu Rev Pharmacol Toxicol (2013) 53(1):401–26. doi: 10.1146/annurev-pharmtox-011112-140320 PMC4680839 23294312

[58] Gao L Mann GE. Vascular NAD(P)H oxidase activation in diabetes: a double-edged sword in redox signalling. Cardiovasc Res (2009) 82(1):9–20. doi: 10.1093/cvr/cvp031 19179352

[59] Zhang Y Tao X Yin L Xu L Xu Y Qi Y. Protective effects of dioscin against cisplatin-induced nephrotoxicity via the microRNA-34a/sirtuin 1 signalling pathway: dioscin protects against CDDP-induced nephrotoxicity. Br J Pharmacol (2017) 174(15):2512–27. doi: 10.1111/bph.13862 PMC5513863 28514495

[60] Cui Y Zhang Z Zhou X Zhao Z Zhao R Xu X. Microglia and macrophage exhibit attenuated inflammatory response and ferroptosis resistance after RSL3 stimulation via increasing Nrf2 expression. J Neuroinflammation (2021) 18(1):249. doi: 10.1186/s12974-021-02231-x 34717678 PMC8557003

[61] Ji H Yi Q Chen L Wong L Liu Y Xu G. Circulating miR-3197 and miR-2116-5p as novel biomarkers for diabetic retinopathy. Clinica Chimica Acta (2020) 501:147–53. doi: 10.1016/j.cca.2019.10.036 31678272

[62] Li Z Dong Y He C Pan X Liu D Yang J. RNA-Seq revealed novel non-proliferative retinopathy specific circulating MiRNAs in T2DM patients. Front Genet (2019) 10:531. doi: 10.3389/fgene.2019.00531 31275351 PMC6593299

[63] Azuma K Koumura T Iwamoto R Matsuoka M Terauchi R Yasuda S. Mitochondrial glutathione peroxidase 4 is indispensable for photoreceptor development and survival in mice. J Biol Chem (2022) 298(4):101824. doi: 10.1016/j.jbc.2022.101824 35288190 PMC8980337

[64] Sun Y Zheng Y Wang C Liu Y. Glutathione depletion induces ferroptosis, autophagy, and premature cell senescence in retinal pigment epithelial cells. Cell Death Dis (2018) 9(7):753. doi: 10.1038/s41419-018-0794-4 29988039 PMC6037763

[65] Liu C Sun W Zhu T Shi S Zhang J Wang J. Glia maturation factor-β induces ferroptosis by impairing chaperone-mediated autophagic degradation of ACSL4 in early diabetic retinopathy. Redox Biol (2022) 52:102292. doi: 10.1016/j.redox.2022.102292 35325805 PMC8942824

[66] Tang X Li X Zhang D Han W. Astragaloside-IV alleviates high glucose-induced ferroptosis in retinal pigment epithelial cells by disrupting the expression of miR-138-5p/Sirt1/Nrf2. Bioengineered (2022) 13(4):8238–53. doi: 10.1080/21655979.2022.2049471 PMC9162003 35302431

[67] Wu Y Lee S Bobadilla S Duan SZ Liu X. High glucose-induced p53 phosphorylation contributes to impairment of endothelial antioxidant system. Biochim Biophys Acta (BBA) - Mol Basis Disease (2017) 1863(9):2355–62. doi: 10.1016/j.bbadis.2017.06.022 PMC5939960 28673515

[68] Luo EF Li HX Qin YH Qiao Y Yan GL Yao YY. Role of ferroptosis in the process of diabetes-induced endothelial dysfunction. WJD (2021) 12(2):124–37. doi: 10.4239/wjd.v12.i2.124 PMC7839168 33594332

[69] Miotto G Rossetto M Di Paolo ML Orian L Venerando R Roveri A. Insight into the mechanism of ferroptosis inhibition by ferrostatin-1. Redox Biol (2020) 28:101328. doi: 10.1016/j.redox.2019.101328 31574461 PMC6812032

[70] Louandre C Ezzoukhry Z Godin C Barbare JC Mazière JC Chauffert B. Iron-dependent cell death of hepatocellular carcinoma cells exposed to sorafenib: iron-dependent cytotoxicity of sorafenib. Int J Cancer (2013) 133(7):1732–42. doi: 10.1002/ijc.28159 23505071

[71] Dixon SJ Patel DN Welsch M Skouta R Lee ED Hayano M. Pharmacological inhibition of cystine–glutamate exchange induces endoplasmic reticulum stress and ferroptosis. eLife (2014) 3:e02523. doi: 10.7554/eLife.02523 24844246 PMC4054777

[72] Dong X Yang L. Inhibition of fatty acid binding protein 4 attenuates gestational diabetes mellitus. Prostaglandins Leukotrienes Essential Fatty Acids (2020) 161:102179. doi: 10.1016/j.plefa.2020.102179 32977290

[73] Fan X Xu M Ren Q Fan Y Liu B Chen J. Downregulation of fatty acid binding protein 4 alleviates lipid peroxidation and oxidative stress in diabetic retinopathy by regulating peroxisome proliferator-activated receptor γ-mediated ferroptosis. Bioengineered (2022) 13(4):10540–51. doi: 10.1080/21655979.2022.2062533 PMC9161966 35441580

[74] Zhang J Qiu Q Wang H Chen C Luo D. TRIM46 contributes to high glucose-induced ferroptosis and cell growth inhibition in human retinal capillary endothelial cells by facilitating GPX4 ubiquitination. Exp Cell Res (2021) 407(2):112800. doi: 10.1016/j.yexcr.2021.112800 34487731

[75] Shen H Gong Q Zhang J Wang H Qiu Q Zhang J. TRIM46 aggravated high glucose-induced hyper permeability and inflammatory response in human retinal capillary endothelial cells by promoting IκBα ubiquitination. Eye Vis (2022) 9(1):35. doi: 10.1186/s40662-022-00305-2 PMC9443035 36064447

[76] Zhang C Hu J Yu Y. CircRNA is a rising star in researches of ocular diseases. Front Cell Dev Biol (2020) 8:850. doi: 10.3389/fcell.2020.00850 33015046 PMC7494781

[77] Zhu Z Duan P Song H Zhou R Chen T. Downregulation of circular RNA PSEN1 ameliorates ferroptosis of the high glucose treated retinal pigment epithelial cells via miR-200b-3p/cofilin-2 axis. Bioengineered (2021) 12(2):12555–67. doi: 10.1080/21655979.2021.2010369 PMC8809929 34903141

[78] Lenaers G Neutzner A Le Dantec Y Jüschke C Xiao T Decembrini S. Dominant optic atrophy: culprit mitochondria in the optic nerve. Prog Retinal Eye Res (2021) 83:100935. doi: 10.1016/j.preteyeres.2020.100935 33340656

[79] Mailloux RJ. Mitochondrial antioxidants and the maintenance of cellular hydrogen peroxide levels. Oxid Med Cell Longevity (2018) 2018:1–10. doi: 10.1155/2018/7857251 PMC6051038 30057684

[80] Pickles S Vigié P Youle RJ. Mitophagy and quality control mechanisms in mitochondrial maintenance. Curr Biol (2018) 28(4):R170–85. doi: 10.1016/j.cub.2018.01.004 PMC7255410 29462587

[81] Perrone L Devi TS Hosoya K Terasaki T Singh LP. Thioredoxin interacting protein (TXNIP) induces inflammation through chromatin modification in retinal capillary endothelial cells under diabetic conditions. J Cell Physiol (2009) 221(1):262–72. doi: 10.1002/jcp.21852 19562690

[82] Yumnamcha T Devi TS Singh LP. Auranofin mediates mitochondrial dysregulation and inflammatory cell death in human retinal pigment epithelial cells: implications of retinal neurodegenerative diseases. Front Neurosci (2019) 13:1065. doi: 10.3389/fnins.2019.01065 31649499 PMC6795687

[83] Lee JY Kim WK Bae KH Lee SC Lee EW. Lipid metabolism and ferroptosis. Biology (2021) 10(3):184. doi: 10.3390/biology10030184 33801564 PMC8000263

[84] Pallardó FV Pagano G Rodríguez LR Gonzalez-Cabo P Lyakhovich A Trifuoggi M. Friedreich ataxia: current state-of-the-art, and future prospects for mitochondrial-focused therapies. Trans Res (2021) 229:135–41. doi: 10.1016/j.trsl.2020.08.009 32841735

[85] Liu J Feener EP. Plasma kallikrein-kinin system and diabetic retinopathy. Biological Chemistry (2013) 394(3):319–28. doi: 10.1515/hsz-2012-0316 PMC4844060 23362193

[86] Bates DO Curry FE. Vascular endothelial growth factor increases microvascular permeability via a Ca(2+)-dependent pathway. Am J Physiology-Heart Circulatory Physiol (1997) 273(2):H687–94. doi: 10.1152/ajpheart.1997.273.2.H687 9277485

[87] Liu Y Zhang Z Yang J Wang J Wu Y Zhu R. lncRNA ZFAS1 positively facilitates endothelial ferroptosis via miR-7-5p/ACSL4 axis in diabetic retinopathy. Oxid Med Cell Longevity (2022) 2022:1–17. doi: 10.1155/2022/9004738 PMC9453005 36092160

